# Interaction of Thiol Antioxidants with α,β-Unsaturated Ketone Moiety: Its Implication for Stability and Bioactivity of Curcuminoids

**DOI:** 10.3390/molecules28237711

**Published:** 2023-11-22

**Authors:** Bo Hyun Lee, Eiseul Song, Jungil Hong

**Affiliations:** 1 Department of Physiology, College of Medicine, Gyeongsang National University, Jinju 52727, Republic of Korea; blee@gnu.ac.kr; 2 Department of Food Science and Technology, College of Natural Science, Seoul Women’s University, 621, Hwarangro, Nowon-gu, Seoul 01797, Republic of Korea; song2busut@swu.ac.kr

**Keywords:** curcumin, curcuminoid, α,β-unsaturated carbonyl group, demethoxycurcumin, bisdemethoxycurcumin, *N*-acetylcysteine, glutathione, thiol antioxidant

## Abstract

Many biological functions of curcumin have been reported. As certain bioactivities of curcumin are eliminated by antioxidants, reactive oxygen species generated by curcumin have been suggested as a relevant mechanism. In the present study, the effects of different types of antioxidants on the stability and bioactivities of curcumin were analyzed. High concentrations (>4 mM) of thiol antioxidants, including *N*-acetylcysteine (NAC), glutathione (GSH), and β-mercaptoethanol, accelerated the decomposition of curcumin and other curcuminoids; the submillimolar levels (<0.5 mM) of GSH and NAC rather improved their stability. Ascorbic acid or superoxide dismutase also stabilized curcumin, regardless of their concentration. The cellular levels and bioactivities of curcumin, including its cytotoxicity and the induction of heme oxygenase-1, were significantly reduced in the presence of 8 mM of GSH and NAC. The effects were enhanced in the presence of submillilmolar GSH and NAC, or non-thiol antioxidants. The present results indicate that antioxidants with a reduced thiol group could directly interact with the α,β-unsaturated carbonyl moiety of curcuminoids and modulate their stability and bioactivity.

## 1. Introduction

Curcumin, also known as diferuloylmethane, is composed of two molecules of ferulic acid and is the primary yellow pigment found in turmeric rhizomes (*Curcuma longa* Linn). Curcumin has been used both as a coloring agent and a traditional remedy for various ailments throughout India and Southeast Asia [1,2]. The biological activities of curcumin have been extensively studied. These activities include antioxidant [3,4,5], anti-cancer [6,7,8], and anti-inflammatory [3,9,10] properties. Various molecular mechanisms have been reported to be involved in the bioactive properties of curcumin. Curcumin scavenges 2,2-diphenyl-1-picrylhydrazyl (DPPH), 2,2′-azino-bis (3-ethylbenzothiazoline-6-sulfonic acid) (ABTS), superoxide anion radicals, and nitric oxide [11,12,13]. Cell shrinkage, chromatin condensation, DNA fragmentation, and the activation of caspase in curcumin-treated cells were observed to induce apoptosis, or programmed cell death [14,15]. Apoptosis induction in several types of cancer cells [16] is suggested to be caused by the rapid generation of reactive oxygen species (ROS) from curcumin in a cell culture system. It was also observed that curcumin inhibits the activation of activator protein-1 (AP-1) and nuclear factor-κB (NF-κB) signaling [17,18]. Moreover, curcumin treatment was found to induce the expression of heme oxygenase-1 (HO-1) [19,20,21] and growth arrest and DNA damage-inducible (GADD) genes [22,23]. It has been shown that curcumin is able to activate antioxidant genes through the nuclear factor erythroid 2-related factor 2 (Nrf2) pathways, providing protective effects [24]. When Nrf2 is activated, it dissociates from Keap1 and moves to the nucleus, leading to the activation of HO-1 [25].

**Figure 1:**
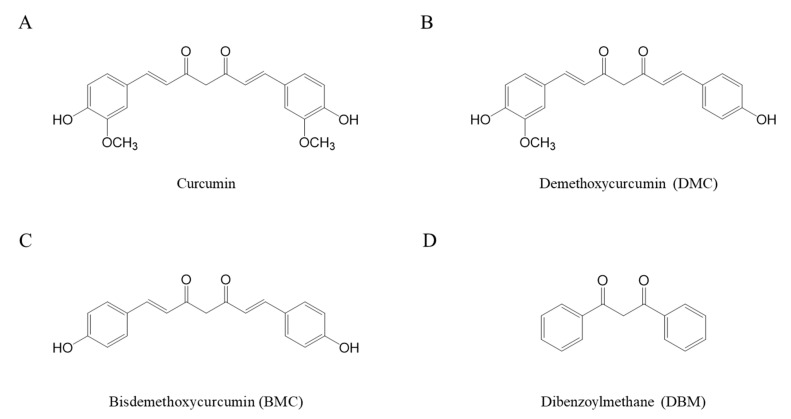
Structures of curcumin (**A**), DMC (**B**), BMC (**C**), and DBM (**D**) used in the present study.

Curcumin contains a bis-α,β-unsaturated group with an electrophilic carbon center, a diketone moiety, and two hydroxyl methoxyphenyl groups (Figure 1A) [19,26]. Curcuminoids that contain a similar structure include demethoxycurcumin (DMC) (Figure 1B) and bisdemethoxycurcumin (BMC) (Figure 1C) [27]. Both DMC and BMC are naturally occurring curcumin analogues that lack one or two methoxy groups on the ring structure of curcumin, respectively [28]. These two compounds are also reported to have a potential role in health benefit effects such as antioxidant activity [27,29,30]. Curcuminoids are not chemically stable due to their highly reactive functional groups. Their chemical stability can be greatly affected by a variety of physical and chemical factors, including pH, temperature, and light [31,32,33]. Curcumin is relatively stable under acidic conditions, but its stability is reduced at alkaline pHs [34,35].

Curcumin exhibits both anti- and pro-oxidizing properties [36,37]. ROS are highly reactive molecules derived from oxygen, including superoxide, hydrogen peroxide, hydroxyl radical, and singlet oxygen. Previous studies have shown that the bioactivities induced by curcumin treatment in cells were eliminated in the presence of antioxidants such as *N*-acetylcysteine (NAC) [38,39] and glutathione (GSH) [40]. One suggestion is that the cellular events induced by curcumin occur through the generation of ROS. Yet, the relationship between the cellular events induced by curcumin in an experimental system and ROS-dependent mechanism has not been clarified. Curcumin is a reactive compound that can potentially interact with various types of chemicals, including antioxidants. Therefore, it remains to be determined whether the suppression of curcumin bioactivities by certain antioxidants is a result of ROS elimination or the direct modification of curcumin’s structure by these antioxidants. 

The present study is based on the hypothesis that thiols may interact directly with curcumin. To test this hypothesis, various methods were employed to compare the effects of thiols with non-thiol antioxidants on the stability and bioactivities of curcumin. First, the absorbance of curcumin at 405 nm was measured to determine whether thiols change the yellowish color of curcumin, indicating a change in its chemical stability. The HPLC analysis determined the precise amount of curcumin. If the stability of curcumin changes, it may be detected through variations in curcumin peaks’ sizes. The study investigated curcumin-induced cell toxicity to identify whether thiols could protect against it. The intracellular levels of curcumin and thiols were measured. Lastly, the impact of curcumin on HO-1 expression levels in the presence or absence of thiol antioxidants was analyzed. Additionally, the stability of DMC and BMC was assessed by incubating them with antioxidants, providing insight into potential interaction sites between the antioxidant and curcumin. The present results suggest that thiol groups interact with the a,β-unsaturated carbonyl moiety of curcumin, which may significantly influence the chemical stability and bioactivity of curcumin and related compounds.

## 2. Results and Discussion

### 2.1. Changes in Curcumin Stability by Different Types of Antioxidants

**Figure 2:**
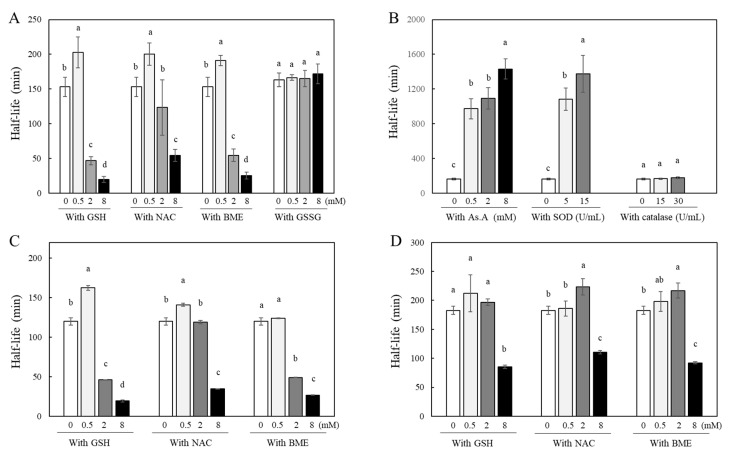
Changes in stability of curcumin by different types of antioxidants. Curcumin (40 μM) was incubated with different concentrations of thiol-related compounds, including GSH, NAC, and BME, as well as GSSG (**A**) or non-thiol antioxidants, including As.A, SOD, and catalase (**B**), in 1 M of phosphate buffer (pH 7.0) at RT. DMC (**C**) and BMC (**D**) (each 40 μM) were also incubated with different thiol antioxidants. At different time points, the absorbance values at 405 nm were measured, and the half-lives (the period required for a 50% color degradation of each curcuminoid) were calculated based on time points of the linear color degradation. Each value represents the mean ± S.D. (*n* = 3–4). Different letters indicate a significant difference (*p* < 0.05) based on one-way ANOVA and the Tukey’s HSD test.

Curcumin is a yellowish hydrophobic compound and is unstable in aqueous systems, causing decolorization. Our previous studies have confirmed that this is due to the instability of curcumin, evidenced by changes in its absorbance at 405 nm [35,41,42]. Since the degree of curcumin’s stability correlates directly with its absorbance at 405 nm, changes in curcumin’s stability in the presence of various antioxidants were measured by detecting absorbance at 405 nm. A solution containing 40 µM of curcumin was incubated at room temperature in the absence or presence of thiol antioxidants, and its half-life was calculated. The incubation of curcumin with higher concentrations of GSH, NAC, and β-mercaptoethanol (BME) such as 2 and 8 mM induced rapid decolorization with similar or significantly shorter half-lives than those of curcumin alone, 153 ± 14 min. This suggests that curcumin became less stable in the presence of high concentrations of thiol antioxidants. In contrast, its incubation with lower concentrations (0.5 mM) of GSH, NAC, and BME showed significantly longer half-lives, 200 ± 16, 191 ± 8, and 202 ± 22 min, respectively, compared to the curcumin alone (Figure 2A). Curcumin clearly showed a biphasic effect in terms of its stability; low concentrations (submillimolar levels at 0.5 mM) of thiol antioxidants improved its stability, whereas higher concentrations (>2 mM) of thiol antioxidants accelerated the degradation of curcumin. Oxidized L-glutathione (GSSG), in which two molecules of GSH are linked by a disulfide bond, is produced through the oxidation of GSH thiol groups in a cellular environment [43]. Interestingly, the stability of curcumin was not affected when incubated with varying concentrations of GSSG, such as 0.5, 2, and 8 mM. The half-lives of curcumin were not significantly different in all the conditions with and without GSSG (Figure 2A).

To determine whether the antioxidant action contributes to the stability of curcumin, the effects of ascorbic acid (AS. A), as a non-thiol antioxidant, and antioxidant enzymes, including superoxide dismutase (SOD) and catalase, on curcumin’s stability were analyzed (Figure 2B). The half-life significantly increased when curcumin was incubated with As.A in a concentration-dependent manner (971 ± 118, 1092 ± 125, and 1430 ± 115 min for 0.5, 2, and 8 mM of As.A, respectively) compared to curcumin alone (163 ± 10 min). When incubated with SOD, curcumin exhibited a stability pattern similar to that when incubated with As.A. At both 5 and 15 U/mL, SOD made curcumin more stable, resulting in longer half-lives. When incubated with catalase, no significant changes in half-lives were observed. These results reveal that non-thiol antioxidants affect curcumin’s stability differently than thiol antioxidants, either causing no significant change or enhancing its stability regardless of concentration.

The stability of both DMC (Figure 2C) and BMC (Figure 2D) was also examined in the absence or presence of thiol antioxidants, GSH, NAC, and BME. When DMC was incubated with a low concentration of GSH or NAC (0.5 mM), the half-lives of curcumin absorbance at 405 nm showed an increase compared to DMC alone, indicating enhanced stability induced by thiol antioxidants. However, when incubated with 0.5 mM of BME, the stability of DMC remained unchanged compared to the stability of DMC alone. GSH, NAC, and BME, at higher concentrations such as 2 and 8 mM, either maintained or decreased the half-lives of DMC compared to DMC alone. In summary, the effect of thiol antioxidants on DMC’s stability is similar to their effect on curcumin. BMC, which lacks two methoxy groups on the ring structure of curcumin, was more stable than curcumin or DMC, with a longer half-life of 182.9 ± 7.1 min. Its incubation with low concentrations of thiol antioxidants (0.5 mM) did not change BMC’s stability significantly. Incubation with 2 mM of NAC or BME but not GSH showed increased half-lives compared to BMC alone, indicating enhanced stability of BMC. However, at the highest concentration of these three thiol antioxidants (8 mM), the half-life of BMC was significantly reduced, indicating an accelerated degradation of BMC’s structure.

While there were minor variances among curcumin, DMC, and BMC, it is evident that thiol antioxidants modulate the chemical stability of curcuminoids with a common a,β-unsaturated carbonyl structure in a biphasic pattern; at a low concentration of thiol-antioxidant, the stability of the curcuminoids improved, but at a higher concentration (8 mM), it decreased consistently. Non-thiol antioxidants solely contribute to the stabilization of curcumin, regardless of concentration, suggesting a possible direct interaction between the thiol group and curcumin. The structural difference between curcumin, DMC, and BMC is the number of methoxy groups on their ring structures; the site of interaction with the thiol group does not appear to be the methoxy phenyl moiety. 

### 2.2. Changes in Curcumin Stability by Antioxidants in a Cell Culture Condition

**Figure 3:**
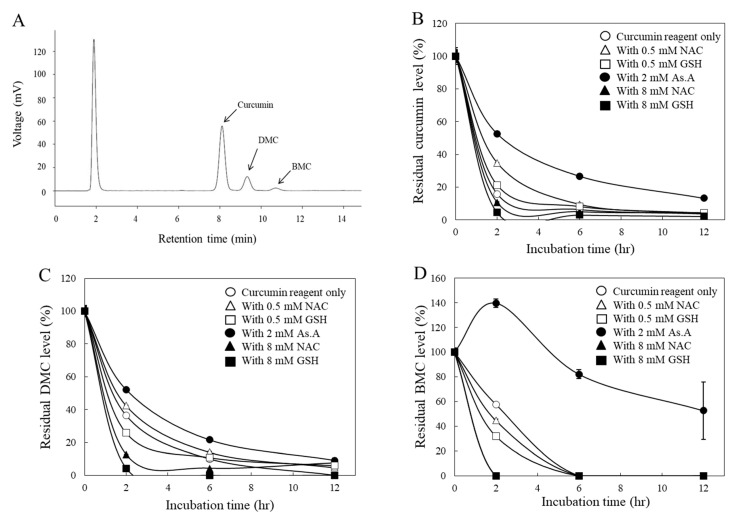
Changes in curcumin levels by different types of antioxidants in a cell culture condition. Chromatograms of the curcumin agent used in the current HPLC system (**A**) are shown. The curcumin reagent (40 μM) containing 79.4, 16.8, and 3.8% of curcumin, DMC, and BMC, respectively, was incubated in the absence or presence of different types of antioxidants under the culture condition with HeLa cells, and the residual levels of curcumin (**B**), DMC (**C**), and BMC (**D**) in the culture medium were analyzed during the 12 h incubation. The two values of filled triangle and filled square in (**D)** are similar and appear to overlap. At each time point, the culture medium was collected, and the amounts of curcuminoids were analyzed by HPLC. Each data point represents the mean ± S.D. (*n* = 3).

Several ROS-dependent mechanisms of curcumin have been reported from studies based on cell culture systems using antioxidants such as NAC [38,39] and GSH [40]. To determine whether antioxidants also affect the stability of curcumin under cell culture conditions, the residual levels of each curcuminoid in the medium were analyzed via HPLC after being used to treat HeLa cells. In the current HPLC system, three distinctive peaks representing curcumin, DMC, and BMC were observed, and their compositions of the curcumin reagent used in this study were 79.4, 16.8, and 3.8% (*w/w/w*), respectively (Figure 3A), which is consistent with other studies [27].

All three curcuminoids, including curcumin (Figure 3B), DMC (Figure 3C), and BMC (Figure 3D), were unstable in the cell culture medium, with noticeable reductions in their concentrations within the initial 2 h. For instance, when curcumin was incubated with a higher concentration (8 mM) of thiol antioxidants, GSH, and NAC, the residual levels of curcumin in the medium decreased more rapidly (Figure 3B). Conversely, a lower concentration (0.5 mM) of them left more curcumin in the medium. Similarly, a non-thiol antioxidant, As.A (8 mM), also resulted in more curcumin remaining in the medium. These results are consistent with the half-life data measured by detecting absorbance at 405 nm (Figure 2A) and the idea that low concentrations of thiol antioxidants increase curcumin stability. 

DMC (Figure 3C) showed similar results to curcumin (Figure 3B), except for the low concentration (0.5 mM) of GSH, which exhibited slightly lower amounts in the medium compared to the DMC alone after a 2 h incubation. Regarding BMC (Figure 3D), neither a low concentration (0.5 mM) of GSH nor NAC increased the residual amounts of BMC in the medium compared to BMC alone after 2 h of incubation. This is also consistent with Figure 2D, which suggests that the low concentration of thiol antioxidants was unable to modulate the stability of BMC. However, As.A (2 mM) stabilized DMC and BMC in the cell culture medium, and the residual levels of DMC and BMC were markedly higher than the control (Figure 3C,D). 

Taken together, both non-thiol antioxidants and a low concentration of thiol antioxidants stabilized curcumin and DMC, whereas high concentrations of thiol antioxidants consistently reduced the stability of all the curcuminoids. In the case of BMC, only the non-thiol antioxidant, As.A, enhanced its stability. Although there was an exception, these findings still suggest that the thiol group of thiol antioxidants may directly interact with curcuminoids. Additionally, the methoxy group in their structure seem unimportant for their interaction with the thiol group, considering that all three curcuminoids showed a similar response to thiol group interactions. 

### 2.3. Changes in Curcumin Cytotoxicity by Antioxidants

**Figure 4:**
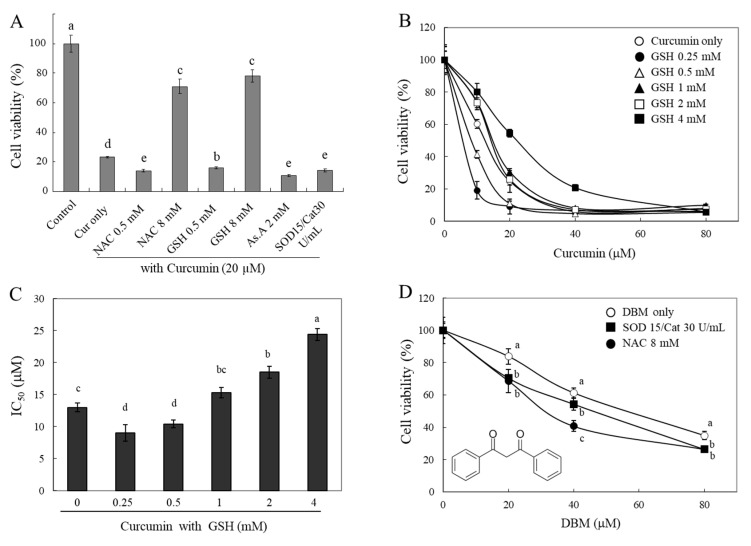
Changes in cytotoxicity of curcumin and DBM by different types of antioxidants. HeLa cells were treated with curcumin (20 μM) in the absence or presence of different types of antioxidants for 24 h, and cell viability was analyzed using the MTT assay (**A**). HeLa cells were also incubated with curcumin in the presence of different concentrations of GSH for 24 h (**B**), and IC_50_ values (concentration induced for 50% inhibition of cell growth) were calculated (**C**). Cytotoxic effects of DBM on HeLa cells were also evaluated in the presence of SOD/catalase (15/30 U/mL) or NAC (8 mM) (**D**). Each value represents the mean ± S.D. (*n* = 8). Different letters indicate a significant difference (*p* < 0.05) based on one-way ANOVA and the Tukey’s HSD test.

If a thiol group of antioxidants directly interacts with curcuminoids and degrades their structures, then their bioactivities, e.g., curcumin-induced cytotoxicity, can be modulated by the thiol antioxidants. To test this hypothesis, we investigated changes in the cytotoxicity of curcumin in the presence of different types of antioxidants (Figure 4A). A strong cytotoxic effect of curcumin (20 µM) on HeLa cells was observed. The presence of thiol antioxidants, GSH and NAC (each 8 mM), was found to significantly reduce the cytotoxic effect of curcumin. Furthermore, there was a significant increase in the cytotoxic effect of curcumin in the presence of 0.5 mM of GSH and NAC, 2 mM of As.A, and 15 and 30 units/mL of SOD/catalase. The results demonstrate that the modulation of curcumin cytotoxicity by thiol antioxidants shows similar biphasic effects to those seen on curcumin’s stability (Figure 2 and Figure 3). Although lower concentrations of thiol antioxidants increased curcumin-induced cell cytotoxicity, higher concentrations of GSH and NAC induced the opposite effect: a reduction in curcumin’s cytotoxicity. These results also reveal that non-thiol antioxidants or antioxidant enzymes can improve curcumin’s stability and increase its cytotoxicity. 

To better understand the action of GSH, we measured the cytotoxicity of curcumin against HeLa cells in the presence of different concentrations of GSH (0.25–4 mM, Figure 4B). Both 0.25 mM and 0.5 mM of GSH potentiated cytotoxicity induced by curcumin on HeLa cells. The cell viabilities were 19 ± 5% and 41 ± 2%, respectively, compared to 10 μM of curcumin alone (60 ± 3%). In contrast, higher concentrations of GSH (2 and 4 mM) decreased the cytotoxicity that was induced by curcumin, and the cell viabilities were 73 ± 3%, and 81 ± 5%, respectively, compared to curcumin alone (60 ± 3%). The IC_50_ was calculated, and it is displayed in Figure 4C. As shown in Figure 4B, the incubation of curcumin with lower concentrations of GSH (0.25 and 5 mM) decreased the IC_50_ to 9.0 ± 1.3 and 10.4 ± 0.6 µM, respectively, compared to curcumin alone (13 ± 0.7 µM). As anticipated, higher concentrations of GSH (2 and 4 mM) caused an increase in the IC_50_ of curcumin up to 24.4 ± 0.9 µM. The concentration-dependent IC_50_ of GSH showed a U-shape, indicating a biphasic effect on curcumin-induced cell cytotoxicity. These findings also indicate that lower concentrations of thiol antioxidants may increase the stability of curcumin, whereas higher concentrations of the thiol group directly impact its structure and result in a decrease in curcumin bioactivities in a thiol-concentration-dependent manner. 

Dibenzoylmethane (DBM) (Figure 1D), which lacks an α,β-unsaturated moiety, was also used for the cytotoxicity evaluation to understand the exact reactive site of curcumin with thiol groups. The cytotoxic effects of DBM were significantly enhanced in the presence of 15 and 30 units/mL of SOD/catalase or 8 mM of NAC (Figure 4D). While 8 mM of NAC significantly reduced the stability and bioactivity of curcumin, this phenomenon was not observed in the case of DBM, and its cytotoxicity was rather enhanced. NAC might be involved only in stabilizing DBM structures; thiol groups are likely reactive with α,β-unsaturated carbonyl structures rather than a diketone moiety. The precise characterization of the chemical modification of curcumin and related α,β-unsaturated diketone derivatives by thiol compounds needs to be explored further. 

### 2.4. Changes in Cellular Levels of Curcumin by Antioxidants

**Figure 5:**
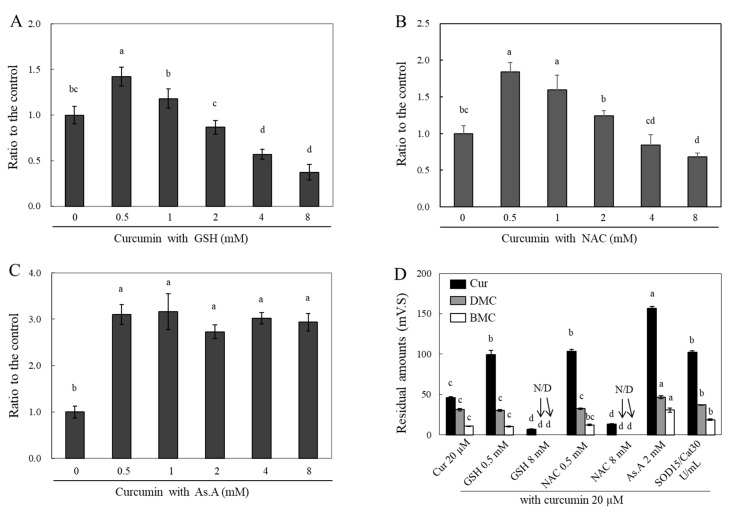
Changes in intracellular levels of curcumin by different types of antioxidants. HeLa cells were treated with curcumin (20 μM) in the presence of different concentrations of GSH (**A**), NAC (**B**), and As.A (**C**) for 4 h. The cells were then lysed using 70% MeOH, and the absorbance of the cell lysates were measured at 405 nm. HeLa cells were also incubated with curcumin (20 μM) in the absence or presence of different types of antioxidants for 2 h. After harvesting cells as described in the materials and methods, the individual curcuminoid levels inside the cells were analyzed via HPLC (**D**). Each value represents the mean ± S.D. (*n* = 3–4). Different letters indicate a significant difference (*p* < 0.05) based on one-way ANOVA and the Tukey’s HSD test.

Curcumin is highly hydrophobic and soluble in lipids. This property allows curcumin to penetrate a plasma membrane [44]. Accordingly, co-treated antioxidants with curcumin were expected to affect the intracellular curcumin level by modulating its stability in media. To answer this question, HeLa cells were treated with varying concentrations of different antioxidants in the presence of curcumin, and then cell lysates were collected to measure the peak absorbance of curcumin at 405 nm (Figure 5A–C) or to conduct an HPLC analysis (Figure 5D). 

Curcumin treatment with different concentrations of GSH (Figure 5A), NAC (Figure 5B), and As.A (Figure 5C) for 4 h was applied to HeLa cells. The cells were subsequently lysed using 70% MeOH, and the absorbances of the cell lysates containing curcumin were measured at 405 nm. The incubation of 0.5 mM of GSH with curcumin resulted in a significant increase in the absorbance value at 405 nm by 1.42 fold in comparison to curcumin alone (Figure 5A). Increasing concentrations of GSH, up to 2 mM, incubated with curcumin led to similar intracellular curcumin levels when compared to incubation with curcumin alone. Cell incubation with curcumin at 4 and 8 mM of GSH caused a significant decrease in absorbance values by 0.57 ± 0.05 and 0.37 ± 0.08 times, respectively, in comparison to curcumin alone. In the case of NAC (Figure 5B), the changes in the absorbance at 405 nm were similarly modulated as with GSH incubation. The incubation of both 0.5 and 1 mM of NAC with curcumin significantly increased the absorbance values to 1.84 and 1.60 fold, respectively, compared to curcumin alone. However, 8 mM NAC incubation with curcumin resulted in a significantly decreased absorbance of 0.68 ± 0.06 fold. After incubating the HeLa cells with 0.5 mM of As.A, the cellular curcumin level increased by 3.10 fold (Figure 5C). Unlike the results of GSH and NAC, the concentrations of As.A up to 8 mM did not alter the absorbance values at 405 nm. No significant change in intracellular curcumin level was observed with different concentrations of As.A. This also confirmed that antioxidants lacking thiol groups do not interact directly with curcumin and only increase its stability. 

The samples for the HPLC analysis were collected by preparing cell lysates according to the methods detailed in the materials and methods section. The intracellular curcumin levels were significantly lower after 2 h of incubation in the presence of 8 mM of GSH and NAC compared to those in the cells treated solely with curcumin (Figure 5D). The intracellular levels of DMC and BMC decreased to almost basal levels in the presence of 8 mM of GSH and NAC. However, the intracellular levels of curcumin increased with the addition of low concentrations of GSH and NAC (0.5 mM each), 2 mM of As.A, and 15/30 units/mL of SOD/catalase by 2.15 and 2.24, 3.39 and 2.21 fold, respectively. The cellular levels of DMC and BMC were significantly increased by 1.50 and 2.86 fold, respectively, in the presence of 2 mM of As.A. They were also increased by 1.18 and 1.75 fold, respectively, in the presence of SOD/catalase compared to the control. The intracellular levels of DMC and BMC did not exhibit noticeable changes at low concentrations (0.5 mM) of GSH and NAC. This could be due to the fact that 0.5 mM of GSH and NAC was less effective for stabilizing BMC and DMC than curcumin, as shown in Figure 2C,D. These results also suggest that high concentrations of thiol antioxidants reduce intracellular curcumin levels by destabilizing its structure, whereas non-thiol antioxidants and low concentrations of thiol antioxidants can enhance its stability and cellular uptake. Accordingly, the stability of intracellular curcumin was similarly affected by different antioxidants as the stability of extracellular curcumin. After the interaction between curcumin and antioxidants that occurred extracellularly in the medium, the remaining curcumin was presumed to enter the cell. Therefore, increasing the stability of curcumin outside of the cell results in more curcumin passing through the membrane and, consequently, higher levels in cell lysates. 

### 2.5. Changes in Intracellular Levels of Thiols

**Figure 6:**
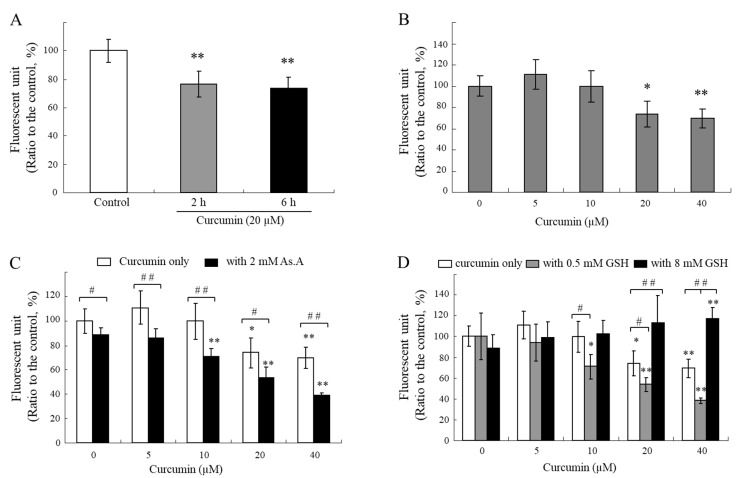
Changes in intracellular thiol levels by curcumin co-incubated with different types of antioxidants. HeLa cells were treated with curcumin only (**A**,**B**) or with As.A (**C**) and GSH (**D**). After 3 h incubation (**B**–**D**), cells were treated with mBBr (40 μM) and then further incubated for 30 min at 37 °C. Changes in fluorescence intensity were detected with an excitation at 365 nm and an emission at 465 nm. Each value represents the mean ± S.D. (*n* = 8). *, ** significantly different from its corresponding control according to Student’s *t*-test (*, *p* < 0.05; **, *p* < 0.01). #, ## significantly different in the same concentration group according to Student’s *t*-test (#, *p* < 0.05; ##, *p* < 0.01).

If curcumin specifically reacts with thiol groups in media, intracellular curcumin is also capable of reacting with thiol compounds in cells. To examine the changes in intracellular thiol levels due to the treatment of curcumin with cells, an assay using monobromobimane (mBBr), a thiol-specific fluorescence probe, was conducted [45]. The intracellular thiol levels were reduced for 2 and 6 h after exposure to curcumin at a concentration of 20 µM (Figure 6A), and its effects seem to be dependent on concentration (Figure 6B). Curcumin might react directly with intracellular thiol-containing biomolecules, such as GSH, or induce oxidative stress that leads to GSH consumption in cells. GSH is considered one of the most abundant endogenous antioxidants. In humans, GSH is present in a high concentration between 1 and 10 mM, which allows it to scavenge ROS [46,47]. 

The treatment of various concentrations of curcumin in the presence of As.A (2 mM) resulted in a greater decrease in the cellular levels of thiols (Figure 6C). A low concentration of GSH (0.5 mM) also lowered the intracellular thiol levels (Figure 6D). The levels of thiols inside cells after the treatment with curcumin in the presence of 8 mM of GSH did not decrease and were even higher than the control levels (Figure 6D). These results suggest that 0.5 mM of GSH or 2 mM of As.A can enhance the stability of curcumin in an extracellular medium, resulting in a higher transfer of curcumin into the cells and more thiols being consumed after direct interaction inside the cells. On the other hand, the stability of curcumin is significantly lower in the presence of 8 mM of GSH in a culture medium, and the level of curcumin transferred into the cells is insufficient to impact the intracellular levels of thiols.

### 2.6. Changes in Curcumin-Induced HO-1 Levels by Antioxidants

**Figure 7:**
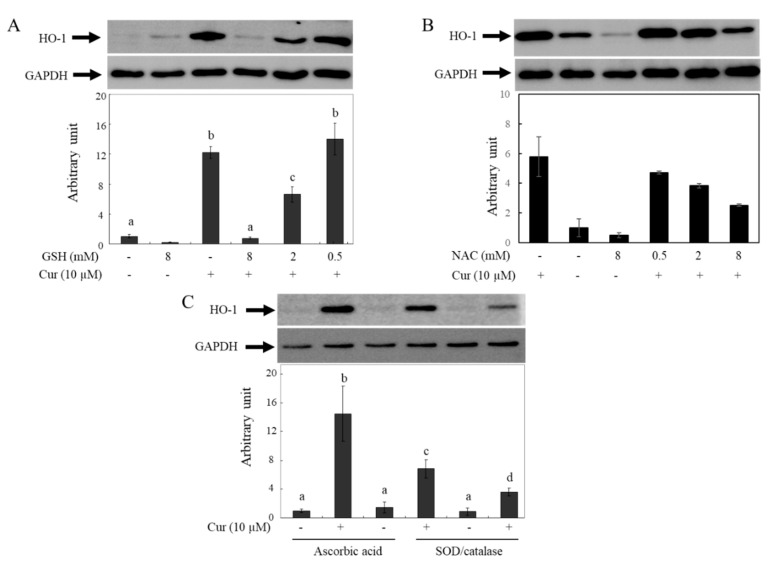
Changes in curcumin-induced HO-1 level by different types of antioxidants. HeLa cells were treated with curcumin in the absence or presence of GSH (**A**), NAC (**B**), and As.A or SOD/catalase (**C**) for 12 h. Western blot analysis was performed on cell lysates containing 20 μg (**A**,**B**) or 10 μg (**C**) of proteins with an antibody against HO-1 (Hsp32). The chemiluminescence was detected and quantified by a luminescent image analyzer. The results are presented as the mean ± S.D. (*n* = 3 in case of (**A**,**C**)) or the mean of duplicates with error bars (**B**). Different letters indicate a significant difference (*p* < 0.05) based on one-way ANOVA and the Tukey’s HSD test.

Previous reports have shown that curcumin induces HO-1 expression and that the effects are caused by ROS generated from curcumin. These reports indicate that HO-1 induction by curcumin is hindered in the presence of GSH or NAC [48]. To confirm whether the decreased bioactivity of curcumin in the presence of GSH or NAC is caused by the removal of ROS or the degradation of curcumin, the effects of different types of antioxidants on HO-1 expression by curcumin were investigated using Western blot analysis (Figure 7). The HO-1 protein levels in HeLa cells increased after incubation with 10 µM of curcumin for 12 hr, but the effects of curcumin were reduced in a concentration-dependent manner by GSH (Figure 7A) and NAC (Figure 7B). Based on our current results, it is hypothesized that the observed phenomenon may be due to curcumin degradation in media by antioxidants containing thiol groups. The induction levels of HO-1 by curcumin increased when co-incubated with non-thiol antioxidants such as As.A and SOD/catalase (Figure 7C). Hence, the lowered induction level of HO-1 by curcumin in the presence of thiol antioxidants such as GSH and NAC cannot be attributed to their ROS-quenching effects; this phenomenon is related to the stability of curcumin. Non-thiol antioxidants may stabilize curcumin, leading to the transfer of more curcumin into cells and stronger physiological responses, including HO-1 induction. Conversely, antioxidants with a thiol group can reduce the bioactivity of curcumin by decreasing its stability in a cell culture system.

Our current results show that thiol groups of antioxidants can directly modify the chemical structure of curcuminoids by targeting the α,β-unsaturated carbonyl moiety. Although low concentrations of thiol compounds from GSH, NAC, and BME stabilize the structure of curcumin through their general antioxidant action, high levels of thiol groups can attack the α,β-unsaturated ketone structure, leading to the rapid decomposition of curcumin and its structurally related compounds, DMC and BMC. The biphasic modulation of curcumin stability by thiol antioxidants could be attributed to the concept of “activation energy”. To facilitate the reaction between the thiol group and curcumin, a relatively high activation energy may be required. Therefore, it appears that a certain concentration of thiols is required for the reaction to proceed. However, low concentrations of thiols are expected to stabilize curcumin due to their general antioxidant activity. Further research is required to establish this precisely.

The α,β-unsaturated compounds function as electrophiles/alkylating agents and are often toxic to the body. However, thiol-containing compounds such as GSH and NAC can provide defense against such toxicity. What products may arise upon curcumin undergoing a direct chemical reaction with thiols? Luis et al. [49] suggested that curcumin forms multiple thiol adducts, including quinone methide, curcumin mono-, and dithioether, after reactive metabolites interact with protein thiols. These findings demonstrate the potential of curcumin to form adducts with thiols, which contribute to curcumin’s chemical and biological effects.

**Figure 8:**
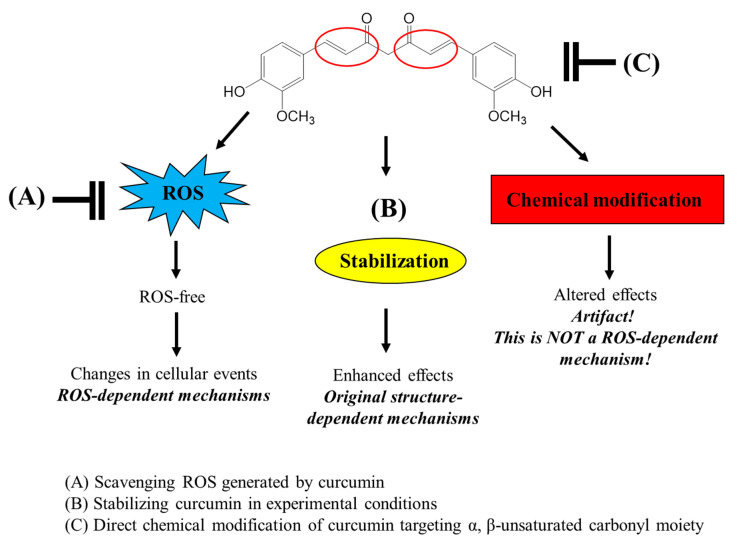
Different roles of thiol antioxidants in modulating bioactivities of curcumin in experimental conditions.

Many studies have used thiol antioxidants, GSH and NAC, to scavenge ROS in experimental systems, particularly using cultured cells, and have attempted to elucidate the ROS-dependent mechanisms of various bioactive compounds. However, the use of these thiol antioxidants should be considered carefully as they can directly modify the structure of the test compounds rather than scavenge ROS. The possible roles of thiol antioxidants in the interaction of curcumin and various bioactive compounds with α,β-unsaturated carbonyl groups in an experimental system with cultured cells are presented in Figure 8. Furthermore, the direct chemical interaction of thiol groups with various compounds containing a α,β-unsaturated carbonyl moiety needs to be carefully considered not only in experimental systems but also in industrial applications such as food and pharmaceuticals. 

## 3. Materials and Methods

### 3.1. Chemicals and Cell Lines

Curcumin (a mixture of curcumin, demethoxycurcumin (DMC), and bisdemethoxycurcumin (BMC), 79.4, 16.8, and 3.8% (*w/w/w*), respectively) was purchased from Acros Organics (Morris Plains, NJ, USA). DMC, BMC, dibenzoylmethane (DBM), *N*-acetyl-L-cysteine (NAC), reduced L-glutathione (GSH), oxidized L-glutathione (GSSG), and β-mercaptoethanol (BME) were purchased from Sigma-Aldrich Chemical Co. (St. Louis, MO, USA). A HO-1 (Hsp32) polyclonal antibody and GAPDH monoclonal antibody (1D4) were obtained from Assay Designs (Ann Arbor, MI, USA). 3-(4,5-dimethylthiazol-2-yl)-2,5-diphenyltetrazolium bromide (MTT) was from Amresco Inc. (Solon, OH, USA). All other chemicals were purchased from Sigma-Aldrich Chemical Co. HeLa human cervical carcinoma cells were obtained from the American Type Culture Collection (Manassas, VA, USA). Cells were maintained in Dulbecco’s modified Eagle’s medium (DMEM) supplemented with 10% fetal bovine serum, 100 unit/mL penicillin, and 0.1 mg/mL streptomycin at 37 °C in 95% humidity and 5% CO_2_. 

### 3.2. Determination of Curcumin Stability with Different Types of Antioxidants

For analyzing changes in chemical stability, curcumin, DMC, or BMC in 1 M phosphate buffer (pH 7.0) with 0.5% dimethyl sulfoxide (DMSO) as the vehicle was incubated at room temperature (RT) with or without different antioxidants. At different time points, changes in the absorbance at 405 nm were detected using a microplate reader (Triad LT, Dynex Technologies Inc., Chantilly, VA, USA).

### 3.3. Analysis of Residual Levels of Curcumin in Media and Cells

HeLa cells were seeded in a 24-well plate and incubated for 24 h. The cells were then treated with curcumin, with or without the addition of GSH, NAC, and As.A, for durations of 2, 6, and 12 h. At each time point, 500 µL of media was collected, and to this, 5 µL of 10 N HCl was added to stabilize the curcumin. An additional 500 µL of ethyl acetate was added to this mixture, followed by an intense 30 s vortex mixing. The mixtures were then centrifuged at 10,000× *g* for 5 min, and then the organic solvent layer was collected. The extraction step was repeated twice. The resultant organic phases were dried using a vacuum centrifuge (NB-503CIR, N-Biotec, Inc., Seoul, Republic of Korea), and the dried extracts were dissolved in the HPLC mobile phase solvent for the HPLC analysis. To analyze the cellular uptake of curcumin, HeLa cells were seeded in a 12-well plate. When the cells reached ~80% confluency, the cells were treated with 20 µM of curcumin in serum-free DMEM media. After 2 or 4 h of incubation, cells were washed 3 times with ice-cold phosphate-buffered saline. Intracellular curcumin was then extracted using 70% MeOH for 30 min, and the cell lysates were centrifuged at 10,000× *g* for 10 min at 4 °C. The supernatant was analyzed at 405 nm using a microplate reader and used for the HPLC analysis. 

### 3.4. HPLC Analysis

The amounts of curcumin and curcuminoids in the media and inside the cells were analyzed with an HPLC equipped with a L-6200 intelligent pump (Hitachi, Ltd., Tokyo, Japan) and an UV-975 UV/vis detector (Jasco Co., Tokyo, Japan). A Shiseido C18 packed column (150 mm × 4.6 mm, 5 μm particle size) was used. The mobile phase consisted of 60% water containing 1% citric acid and 40% THF adjusted to pH 3.0 with a concentrated KOH solution (*v/v*) (40). The solvent was run isocratically at a flow rate of 1.0 mL/min. The peak was achieved at 420 nm, and the injection volumes were 20 μL. The operating condition is described in Table 1. 

### 3.5. Evaluation of Cell Cytotoxic Properties

The cytotoxic effects of curcumin on HeLa cells were determined using the MTT assay. The cells were seeded in 96-well plates at a density of 10^4^ cells per well and treated the next day with curcumin or DBM in the absence or presence of different types of antioxidants. After 24 h, the compound-containing medium was removed, and 100 mL of fresh medium containing 0.5 mg/mL MTT was added to each well. The cells were further incubated at 37 °C for 1–2 h. The medium was then replaced with 100 mL of DMSO, and absorbance was measured at 550 nm using a microplate reader (Triad LT).

### 3.6. Measurement of Intracellular Thiol Levels

The levels of intracellular thiols were measured using a cell-permeable thiol-staining reagent called monobromobimane (mBBr), as described by Kong et al. with slight modifications [43]. HeLa cells were seeded in 96-well plates with a density of 10^4^ cells per well. After 24 h, the cells were treated with curcumin in the absence or presence of various types of antioxidants. Following different incubation times, the medium was removed and washed with PBS. The cells were incubated with 100 μL of a 40 μM solution of mBBr for 30 min at 37 °C. After that, the mBBr solution was removed, and 0.2 N NaOH was added to lyse the cells. The fluorescence intensity from thiols reacting with mBBr was measured using a fluorescent microplate reader (Triad LT). The excitation and emission wavelengths were 365 nm and 465 nm, respectively.

### 3.7. Western Blot

HeLa cells were plated into a 6-well plate. After 24 h, the cells were treated with curcumin in the absence or presence of antioxidants for 12 h. The cells were washed with ice-cold PBS twice and lysed with a cell lysis buffer (1 mM PMSF, 150 mM NaCl, 1 mM Na_2_EDTA, 1 mM EGTA, 1% Triton, 2.5 mM sodium pyrophosphate, 1 mM β-glycerophosphate, 1 mM Na_3_VO_4_, 1 mg/mL leupeptin, in 20 mM Tris-HCl, pH 7.5). The cell lysate was centrifuged at 10,000× *g* for 10 min at 4 °C. The supernatant containing 10 or 20 mg of protein was loaded onto 8% SDS polyacrylamide gel. After electrophoresis, the proteins were transferred onto a PVDF membrane. After blocking, the blots were probed with a human heme oxygenase-1 (Hsp32) polyclonal antibody in 5% non-fat milk and 0.05% tween-20 in PBS. After incubation overnight at 4 °C, the primary antibody was probed with peroxidase-conjugated goat anti-rabbit IgG for 3 h. After washing with 0.05% TBST four times, a Western blotting luminol reagent (Santa Cruz Biotechnology, Inc., Santa Cruz, CA, USA) was added to the blotted proteins, and chemiluminescence was detected using a luminescent image analyzer (LAS-4000 mini, Fujifilm, Tokyo, Japan). The protein concentrations in the cell lysates were determined using a BCA protein assay kit (Pierce, Rockford, IL, USA).

### 3.8. Data Analysis

All values represent the mean ± standard deviation (SD). Each experiment was repeated at least 3 times in triplicate, with some exceptions. Statistical differences were evaluated using the Student’s *t*-test or a one-way ANOVA in the SPSS program (IBM SPSS Statistics 24, SPSS Inc. Chicago, IL, USA), and the Tukey’s honestly significant difference (HSD) test (*p* < 0.05) was used for comparing multiple results.

## 4. Conclusions

Antioxidants that possess thiol groups, such as GSH, NAC, and BME have the capability to modify the structure of curcuminoids by specifically targeting their α,β-unsaturated carbonyl group. In the case of non-thiol antioxidants such as As.A, GSSG, and SOD/catalase, curcumin’s stability either undergoes no significant change or is enhanced. The effects of thiol groups on curcuminoids manifest in a concentration-dependent manner: at concentrations equal to or below 0.5 mM, thiols stabilize curcuminoid through antioxidant activities, but at levels exceeding 4 mM, they induce decomposition via direct chemical interactions. Consequently, when employing GSH and NAC to modulate ROS in cell culture systems, one must exercise caution, as thiol compounds might modify the structures of test molecules through direct chemical reactions rather than by ROS scavenging. Such interactions have profound implications for both academic research and industrial applications.

## Figures and Tables

**Table 1:** Operating condition of HPLC for curcuminoid analysis.

Instrument	L-6200 (Hitachi, Ltd. Tokyo, Japan)
Column	packed column C18 (Shiseido, 4.6 mm ID × 150 mm × 5 µm)
Detector	UV detector (UV-975, Jasco, Tokyo, Japan)
Flow rate	1 mL/min
Injection volume	20 µL
Mobile phase	40% THF: 60% water containing 1% citric acid
	(*v/v/v*, adjust concentrated KOH, pH 3)

## Data Availability

Data available from the corresponding author upon reasonable request.
